# The gut-heart axis: unveiling the roles of gut microbiota in cardiovascular diseases

**DOI:** 10.3389/fcvm.2025.1572948

**Published:** 2025-05-26

**Authors:** Yuan Zhang, Huimin Wu, Mu Jin, Guirong Feng, Sheng Wang

**Affiliations:** ^1^ Department of Anesthesiology, Beijing Anzhen Hospital, Capital Medical University, Beijing, China; ^2^ Department of Anesthesiology, International Mongolian Hospital of Inner Mongolia, Hohhot, Inner Mongolia, China; ^3^ Linzhi People’s Hospital, Linzhi, Tibet, China

**Keywords:** gut microbiota, cardiovascular diseases, heart failure, metabolites, microbiota composition

## Abstract

The gut microbiome refers to the collective genomes of the approximately 1,000–1,150 microbial species found in the human gut, called the gut microbiota. Changing the gut microbiota composition has been shown to affect cardiovascular health significantly. Numerous studies have demonstrated the part that gut microbiota and its metabolites play in the development and course of several illnesses, including colon cancer, heart failure, stroke, hypertension, and inflammatory bowel disease. With cardiovascular diseases responsible for more than 31% of all fatalities globally, conditions like hypertension, atherosclerosis, and heart failure are serious global health issues. Developing preventive measures to fight cardiovascular diseases requires understanding how the gut microbiota interacts with the cardiovascular system. Understanding the distinctive gut microbiota linked to cardiovascular diseases has been made possible by microbial sequencing analysis. The gut microbiota and cardiovascular diseases are closely related, and more profound knowledge of this association may result in treatment strategies and broad guidelines for enhancing cardiovascular health through gut microbiome modification. This review summarizes the role of gut microbiota in cardiovascular diseases, highlighting their influence on disease progression and potential therapeutic implications.

## 1 Introduction

It is estimated that between 1,000 and 1,150 different types of microorganisms reside in the human gut (1). These groups are referred to as “gut microbiota,” and “gut microbiomes” are the collective term for all of the genomes of microorganisms in the gut, including their DNA sequences and other genetic data (2). A small number of phyla, such as *Firmicutes*, *Bacteroidetes*, *Proteobacteria*, *Actinobacteria*, and *Verrucomicrobia*, dominate the gut microbiota in healthy people, which maintains a very consistent composition (3). There is growing evidence that altering the composition of the gut microbiota impacts the cardiovascular phenotypes of the host (4–6).

Numerous research conducted in recent years have shown how the gut microbiota and its metabolites influence the development of both cardiovascular and non-cardiovascular diseases in the host, including inflammatory bowel disease, colon cancer, hypertension, heart failure, and stroke (7). One of the leading causes of death and morbidity globally is cardiovascular disease (CVD). It is the primary cause of death worldwide and includes heart failure, atherosclerosis, and hypertension (8). Atherosclerotic cardiovascular disease is now widely acknowledged as a serious global health hazard, accounting for almost 31% of all fatalities worldwide (9). Finding potential preventive measures is crucial to halting the onset and progression of CVD. Numerous details regarding the existence of distinctive gut microbiota linked to CVDs have been made available by microbial sequencing study (10–12). Gut microbiota has been linked to peripheral artery disease (PAD), especially in diabetic individuals, in addition to its well-established roles in hypertension and coronary artery disease. Atherosclerosis and vascular problems in PAD are accelerated by endothelial dysfunction, oxidative stress, and chronic inflammation, all of which are exacerbated by dysbiosis in the gut microbiota. Furthermore, metabolites originating from the gut, like trimethylamine-N-oxide (TMAO), have been connected to vascular dysfunction and enhanced platelet aggregation, which exacerbates ischemia conditions in PAD. The importance of microbiota in diabetic PAD was emphasized by Biscetti et al., who proposed that microbial manipulation might be a novel treatment approach to enhance vascular outcomes in these patients (13). A growing body of research has demonstrated a substantial correlation between CVDs and gut microbiota (14, 15). By better understanding the relationship between the gut microbiota and the cardiovascular system, we may be able to develop general guidelines and therapeutic strategies that support the gut microbiota's cardio-protective function. In this review, we summarize the role of gut microbiota in cardiovascular diseases.

## 2 Gut microbiota mechanisms of action in cardiovascular diseases

**Figure 1:**
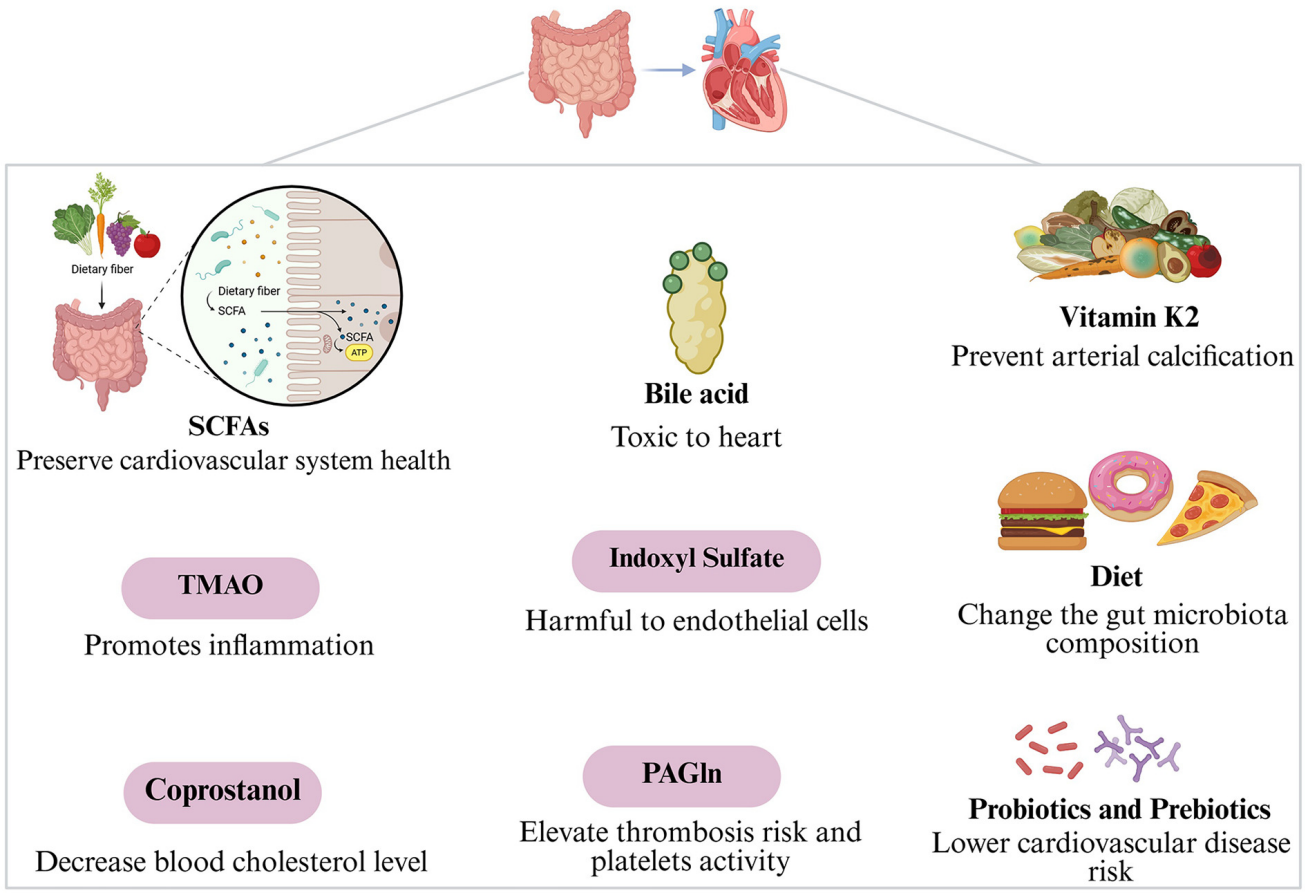
Gut microbiota mechanisms of action in cardiovascular diseases.

The gut microbiota might influence cardiovascular disorders through metabolites like short-chain fatty acids, trimethylamine-N-oxide, coprostanol, bile acid, indoxyl sulfate, phenylacetylglutamine, and vitamin K2 (Figure 1).

### 2.1 Short-chain fatty acid production

The metabolites acetate, butyrate, and propionate are categorized as short-chain fatty acids (SCFAs) and are produced when intestinal bacteria ferment the indigestible polysaccharides (16). SCFAs diffuse across the intestinal mucosa, enter the bloodstream through the portal system at low millimolar concentrations, and interact with G protein-coupled receptors (GPCRs) on the plasma membranes of various target cells throughout the mammalian body (17, 18). These fatty acids exhibit a positive correlation with *Eubacterium rectale*, *Alistipes putredinis*, *Bacteroides spp.*, *Roseburia*, and *Faecal prausnitzii* (19). By improving intestinal barrier integrity through increased production of cellular junction proteins and acting as energy substrates for epithelial cells, SCFAs positively affect the gastrointestinal tract. Furthermore, they affect inflammation and metabolic processes. They can reduce oxidative stress, show anti-inflammatory and anti-tumorigenic properties, alter glycemic control and lipid metabolism, and alter the release of inflammatory cytokines and chemokines (20, 21).

The importance of SCFAs in preserving cardiovascular system health is suggested by their notable effects against various inflammatory illnesses, such as Coronary artery disease (CAD) and hypertension (22, 23). The amount of dietary fiber consumed and the composition of the gut flora's SCFA-producing bacteria control how much SCFA is produced. Accordingly, changes in the composition of the gut microbiota are a significant factor influencing the levels of SCFAs, thereby promoting the development of CVD linked to inflammation (24). There are two main ways that SCFAs might demonstrate their anti-inflammatory properties. The first is that SCFAs are known to inhibit histone deacetylase (HDAC). When HDAC inhibits the histone acetylation process, the chromatin structure decondenses, increasing gene expression and promoting the development of regulatory T cells to generate anti-inflammatory cytokines like interleukin 10 (IL-10) (22, 25). However, butyrate is a negative modulator of inflammation, and its anti-inflammatory action is achieved by inhibiting HDAC, which typically controls innate immunity pathways, regulating the differentiation of myeloid cells and the inflammatory response mediated by the expression of genes induced by toll-like receptors (TLR) and interferon (IFN) (26, 27). Thus, butyrate inhibits HDAC to limit the synthesis of pro-inflammatory cytokines like interferon-γ (IFN-γ), IL-12, and tumor necrosis factor-α (TNF-α). This increases monocytes' *in vitro* production of IL-10, which has anti-inflammatory effects (28). Second, it is found that SCFAs contribute to anti-inflammatory responses through the activation of specific G protein-coupled receptors (GPCRs). The intestinal epithelial cells and almost all immune cell types express GPCRs, such as GPCR41, GPCR43, and GPCR109A, which SCFAs bind to (29). SCFA binding to the GPCR can trigger several intracellular signaling cascades, including nuclear transcription, enzyme activation, and cell membrane ion transport (30). This binding protects the gut from inflammation by promoting regulatory T-cell differentiation to produce IL-10 and enhancing intestinal barrier integrity through the NLR family pyrin domain containing 3 (NLRP3) inflammasomes. These inflammasomes generate IL-19, which enhances epithelial integrity by improving the function of tight junction proteins (29, 31).

Butyrate regulates blood pressure by activating the GPCR41 and vasorelaxing the blood vessels (32). Butyrate specifically lowers diastolic blood pressure in humans. The effects of butyrate on 60 patients with type 2 diabetes mellitus were investigated in a randomized, double-blind trial. The results showed that the diastolic blood pressure of the treatment groups decreased statistically significantly (*P* < 0.05). Additionally, treated patients had higher levels of *Akkermansia muciniphila*, a bacteria with anti-inflammatory properties (33). Propionate activates GPCR41 to cause vasodilation, which lowers blood pressure slightly. The improvement of endothelial dysfunction may be the mechanism for the long-term decrease in blood pressure (34). By preventing the production of cholesterol and moving it to the liver, SCFAs can help reduce serum lipid levels (35). As a result, they have been proposed as a protective factor against the development of CAD. Additionally, there has been a decrease in SCFA-producing bacteria in some CAD cases and the gut dysbiosis of hypertension patients (36–39). Additionally, the intestinal metabolism of cholesterol is influenced by SCFAs. There is an inverse relationship between serum cholesterol levels and the conversion of cholesterol to coprostanol in patients with elevated fecal amounts of SCFAs; the precise mechanisms are still unclear, although this could be because the gut flora is different (40). Although there is growing evidence that short-chain fatty acids are involved in several cardiovascular disorders, more investigation is necessary to fully understand the underlying mechanism and the diverse effects of SCFA supplementation on the cardiovascular system.

### 2.2 Trimethylamine-N-oxide production

TMAO, a risk factor for the development of CAD, is produced by dietary choline, betaine, phosphatidylcholine, lecithin, and L-carnitine (41–44). Choline, phosphatidylcholine, and carnitine-trimethylamine are frequently found in meat, egg yolks, and high-fat dairy products. These substances undergo a two-step modification process: (A) undergo conversion by the gut bacteria into TMA, which is then taken up and transported to the liver via the portal circulation. 36 species with 102 genomes have been reported to create TMA. *Firmicutes*, *Proteobacteria*, and *Actinobacteria* are among the TMA producers; Bacteroidetes do not produce TMA. TMA production has been linked to *firmicutes*, such as *Anaerococcus*, *Clostridium*, *Desulfitobacterium*, *Enterococcus*, *Streptococcus*, and *Proteobacteria*, such as *Pseudomonas*, *Enterobacter*, *Proteus*, *Escherichia*, *Dseulfovibrio*, *Actinobacter*, *Citrobacter*, and *Klebsiella* (45, 46). (B) The flavin-containing monooxygenase (FMO) enzyme, which is expressed by the FMO gene in the liver, kidney, and other tissues, converts the microbiome-derived TMA molecule into TMAO once it enters the host's circulation and reaches hepatocytes (43, 47, 48). Microbiota, age, medicines, gender, and lifestyle all interact to affect TMAO levels (49). The volume of dietary precursor ingestion and the composition of each person's gut microbial flora determine how much TMAO each individual produces (50). Animal products with high concentrations of TMA precursors are a feature of Western diets, altering the gut flora and raising TMAO levels (51).

In contrast to SCFAs, TMAO is a bacterial metabolite that promotes inflammation and has also been linked to the pathophysiology of cardiovascular disease. To cause thrombus development, it works by triggering the immunological and inflammatory responses, upregulating the expression of inflammatory cytokines, and preventing the synthesis of bile acids (52). A higher risk of cardiovascular events and mortality has been associated with elevated TMAO concentrations by 23% and 62%, respectively. A meta-analysis of more than 25,000 participants found that all-cause mortality increased by 7.6% with every 10 μmol/L accumulation of TMAO (53, 54). TMAO may contribute to the pathogenic process of atherosclerosis development by stimulating macrophage migration and its conversion into foam cells. Foam cells have a proatherogenic action from the first lesion generation to the plaque rupture. The biological signature and initial stage of atherogenesis is the buildup of foam cells in artery intima. Most foam cells come from macrophages, which can control the metabolism of lipoproteins (41, 42). TMAO influences cholesterol metabolism and induces the production of foam cells in several ways. High-density lipoprotein (HDL) is a key component of the reverse cholesterol transport (RCT) pathway, which keeps cholesterol metabolism in equilibrium. It stimulates macrophages to release free cholesterol (55). Research revealed that HDL was considerably lower in the plasma of CVD patients with elevated TMAO levels, which prevented the RCT pathway and macrophage accumulation of cholesterol (41). Elevated TMAO levels are associated with increased cardiovascular risks and accelerated atherosclerosis. Additionally, TMAO increases endothelial dysfunction, changes lipid metabolism, and causes platelet hyperactivity by stimulating calcium release from the rough endoplasmic reticulum. Platelet hyperactivity and the consequent development of plaque are caused by TMAO, which also increases cholesterol influx, decreases cholesterol circulation, and blocks the bile acid route (56–58). Choline supplementation raises TMAO levels and enhances platelet aggregation and reactivity in healthy human volunteers (59). Non-culprit plaques show signs of vulnerability in CAD patients with increased TMAO. These include enhanced microvascularization, a higher incidence of thin-cap fibroatheroma, and decreased fibrous cap thickness (10, 60). C-reactive protein (CRP) and endothelial dysfunction with elevated gut permeability are also linked to TMAO (45, 58).

The relationship between elevated TMAO plasma levels and CVD, as well as its association with major adverse cardiac events (MACEs) like myocardial infarction, stroke, heart failure, and cardiovascular death, has been assessed by recent metabolomics investigations (42, 61, 62). The rate of MACEs was strongly correlated with plasma TMAO concentrations over a 3-year follow-up period in individuals who had elective coronary angiography. Even after controlling for conventional risk variables, patients in the highest quartile of circulating TMAO levels had a 2.5-fold increased risk of MACEs compared to those in the lowest (63). Even though numerous investigations have verified the link between TMAO levels and different CAD occurrences, some studies do not find a correlation between TMAO and CAD. One study, for instance, found no correlation between TMAO levels and atherosclerosis in the Framingham Heart Study Offspring group (1,215 participants) or supporting animal research (64, 65). Studies have demonstrated that the use of broad-spectrum antibiotics changed the composition of the gut microbiota and decreased TMAO levels, indicating the significance of the gut microbiota in the metabolism of TMAO, which is consistent with the mandatory role that the gut microbiota performed in TMAO formation (41). Elevated TMAO levels are linked to a greater risk of adverse cardiovascular disease, so further large-scale prospective cohorts are expected to characterize the association, especially the causality in the general population.

### 2.3 Coprostanol production

In addition to TMAO, there are various microbial pathways whose alteration or suppression could impact CVD risk. The gut bacteria's ability to convert cholesterol to coprostanol was initially documented in the 1930s (66). Humans begin producing coporstanol in the second half of their first year of life (67), as well as being sex-dependent, with young women having higher conversion rates than young men (68, 69). The gut converts cholesterol to coprostanol, a non-absorbable sterol removed with feces. Because it is linked to decreased blood cholesterol levels, converting cholesterol to coprostanol has a clinically significant effect (70). One potential method is the conversion of cholesterol to sterol coprostanol by the microbial cholesterol dehydrogenase enzyme (ismA gene) (71, 72). The number of bacteria that metabolize cholesterol was shown to be closely associated with factors that affect cholesterol conversion (73, 74). Intestinal bacteria that can convert cholesterol to coprostanol include *Eubacterium* coprostanoligenes, *Bacteroides* species, *Lactobacillus* species, and *Bifidobacterium* species. IsmA has a role in the oxidation of coprostanol to coprostanone and cholesterol to 4-cholesten-3-one. Interestingly, *Escherichia coli*overexpresses this enzyme, and other microbial species that have IsmA genes have been linked to lower blood cholesterol levels (75). Using animal models, giving hypercholesterolemic rabbits the cholesterol-lowering bacterium Eubacterium coprostanoligenes dramatically lowered their plasma cholesterol levels. The intestinal contents of rabbits-fed microorganisms showed higher than average coprostanol/cholesterol ratios (76). Many studies on the gut's metabolism of cholesterol have been conducted using human models (67, 77–80), and it has been proposed that the coprostanol/cholesterol ratio in human feces and human serum cholesterol have an inverse relationship (79, 81, 82). Coprostanoligenic strain research may be useful in the clinic to modify the microbiota and lower cardiovascular risk (83, 84). Further research is necessary because the precise mechanisms are still unclear.

### 2.4 Bile acid modulation

The main amphipathic, water-soluble byproduct of the breakdown of cholesterol is bile acids (BAs) (85). They have a role in inflammatory bowel disease, gastrointestinal cancer, metabolic disorders, and cardiovascular diseases (86). Since the 1960s, it has been shown that BAs are toxic to the heart. They can induce cardiac remodeling and electrophysiological changes, which can lead to deadly arrhythmias (87, 88). Because the ileum reabsorbs around 95% of the bile acids and the enterohepatic cycle returns them to the liver, bile acids can act as endocrine-like signaling molecules that alter host metabolism and energy homeostasis (89). The primary metabolites of cholesterol in the liver that aid in the absorption of lipids, nutrients, and lipophilic vitamins are bile acids, which are produced by the rate-limiting enzyme cholesterol 7-alpha-hydroxylase (CYP7A1) (90, 91), and also the energy metabolism, regulation of lipids and glucose (92, 93).

Bile salts are created when primary bile acids are coupled to the amino acids taurine or glycine. These salts are then secreted into bile and kept in the gallbladder until released into the small intestine, emulsifying fats and creating micelles that enterocytes can absorb (91). Primary bile acids like cholic acid (CA) and chenodeoxycholic acid (CDCA) are broken down in the gut by bile salt hydrolase (BSH) and the gut microbiota to produce secondary bile acids, including deoxycholic acid (DCA), lithocholic acid (LCA), and ursodeoxycholic acid (UDCA) (91, 94). Except for UDCA and LCA, primarily eliminated in feces, all conjugated and unconjugated bile acids in the lumen can be reabsorbed (95%) and returned to the liver (90). The nuclear receptor farnesoid X receptor (FXR) and the membrane G protein-coupled bile acid receptor Gpbar-1 can be activated by signaling molecules like bile acids in the gut (91, 95). Bile acids can suppress bile acid synthesis through this method (94), which may raise cholesterol levels (96). In the intestine, a wide range of aerobic and anaerobic bacteria (including Gram-negative *Bacteroides*, *Lactobacillus*, *Clostridium*, and *Enterococcus*, as well as Gram-positive *Bifidobacterium*) catalyze the deconjugation of the glycine and taurine moieties of PBA (97). Then, PBA (CA and CDCA) are changed into secondary bile acids (SBA): LCA and DCA, respectively, by bacterial hydroxysteroid dehydrogenase (HSDH) enzymes, which eliminate a hydroxyl group at the 7α position (98). Secondary bile acids (SBAs) have been linked to cardiovascular health and are ligands of the nuclear farnesoid X receptor (FXR). Diet can alter the composition of the gut microbiota and the metabolism of bile acids (99). The gut microbiota can impact how the liver regulates cholesterol metabolism (28, 81) and contribute to the modification of bile acids, which can affect systemic cholesterol levels (100). Bile acids may be a helpful biomarker to predict the severity of CVD, according to cohort studies conducted in various populations (101, 102). Elevated plasma levels of LCA were linked to an increased risk of CHD in a case-control study (103). Patients with CAD had higher fasting serum total BA than those without CAD, according to a survey of 7,438 participants (104). Additional research is necessary to validate and deepen our comprehension of BA's function in modulating the association between gut microbiota and the risk of CVD.

### 2.5 Indoxyl sulfate

Tryptophan, a necessary amino acid and a precursor to various important mediators such as tryptamine, serotonin, melatonin, kynurenines, and nicotinic acid, is converted in the intestines into the molecule indole by gut bacteria (105–107). *Escherichia coli* and other gut bacteria include tryptophanase, which converts a portion of tryptophan obtained from proteins into indole (108). The liver produces the indole metabolite indoxyl sulfate, which enters the bloodstream as a serum molecule coupled to albumin. Usually, indoxyl sulfate is eliminated by the urine. Because indoxyl sulfate is not adequately eliminated in patients with poor renal function, it increases in the blood and negatively impacts the endothelium by causing arterial stiffness and calcification. It is well-recognized that indoxyl sulfate damages various cell types, including vascular endothelial cells (109–112). In endothelial cells, elevated indoxyl levels cause oxidative stress, a pro-inflammatory response, and increased adhesion molecule expression. The pathophysiology of cardiovascular disorders in people with chronic kidney disease (CKD) is influenced by the harmful effects of indoxyl on endothelial cells. How indoxyl affects the pathophysiology of CAD in individuals who do not have renal impairment is still unknown (113). Among dialysis patients with normal renal function, indoxyl sulfate was associated with cardiovascular disease (114).

Serum indoxyl sulfate levels may be a predictive mechanistic biomarker of the severity of coronary artery disease because they have a positive correlation with coronary atherosclerosis scores (115). Indoxyl sulfate causes thrombosis via increasing platelet activity and the body's reaction to collagen and thrombin (116). There is evidence linking an increase of the nephrotoxin indoxyl sulfate in the serum of uremic patients—which is caused by poor renal secretion—to gut microbiota dysbiosis toward a greater abundance of aerobic indole-producing bacteria (such as *Escherichia coli*) (117, 118). Studies by Takayama et al. demonstrated that by altering the composition of the gut microbiota, hemodialysis patients receiving oral treatment with non-indole-producing bacteria (such as *Bifidobacterium*) had a significant decrease in indoxyl sulfate serum levels (118). Another study demonstrated that giving indoxyl sulfate to rats with CKD accelerated the disease's progression and raised the expression of the genes for pro-α1(I) collagen, tissue inhibitor of metalloproteinase (TIMP)-1, and transforming growth factor (TGF)-β1 (119). Additional research has demonstrated that indoxyl sulfate exacerbates atrial fibrillation, cardiomyocyte hypertrophy, and cardiac fibrosis (120, 121). These investigations collectively show that indoxyl sulfate is biologically connected to CVD at both the molecular and cellular levels.

### 2.6 Phenylacetylglutamine

The gut flora of heart failure patients and healthy people varies in terms of both composition and function, according to a number of studies conducted in recent years. Glutamate, which lowers ammonia through the urea cycle bypass pathway, and phenylacetate/phenylbutyrate (phenylacetate precursor) were originally believed to be the only sources of phenylacetylglutamine (PAGln), one of the main toxins associated with chronic kidney illness (122). Recent research, however, has shown that PAGln, which is also produced by intestinal microbes that metabolize the essential amino acid phenylalanine, is regarded as an independent predictor of MACE risk and has high plasma concentrations in patients who have experienced MACE (104, 123). A metabolite called PAGln is produced when the gut bacteria conjugates glutamine and phenylacetate. PAGln has been linked to elevated thrombosis risk and platelet activity (103). *Klebsiella pneumoniae*, *Acinetobacter baumannii*, *Proteus mirabilis*, *Lachnospiraceae*, *Christensenellaceae*, and *Ruminococcaceae.* Among the main microbes that produce phenylacetylglutamine are *Bacteroidetes*, *Firmicutes*, *Proteobacteria*, and *Staphylococcus aureus* (124–127).

High levels of PAGln are independently linked to a higher risk of coronary in-stent restenosis, which is linked to a poorer prognosis for CAD patients (128). Hazen et al. were the first to show a positive association between the gut-derived metabolite PAGln and platelet and thrombosis functions in a nontargeted metabolomics investigation (123). Additionally, elevated PAGln plasma levels are a strong independent predictor of carotid plaque burden, indicating a potential link to atherosclerosis (129). According to a study by Fang et al, increased plasma PAGln levels and improved microbiota-derived PAGln synthesis-related functions were linked to in-stent stenosis and hyperplasia in CAD patients. To prevent stent stenosis in patients with CAD, an intervention that targets gut bacteria may be a promising approach (130).

### 2.7 Vitamin K2

There are two main types of vitamin K: phylloquinones (vitamin K1, VK1) and menaquinones (vitamin K2, VK2). Four isoprenoid residue side chains make up the single chemical VK1. However, the side chains of VK2 range in length from four to thirteen isoprene residues (131). Plants are the exclusive source of VK1, but gram-positive bacteria in the human gastrointestinal system create a number of congeners (menaquinone-5–menaquinone-13, MK5–MK13) that make up VK2 (131, 132). Vitamin K2 is an isoform of vitamin K and a cofactor that helps carboxylate several proteins, including matrix Gla-protein (MGP) (133). Fibroblasts, endothelial cells, vascular smooth muscle cells, and chondrocytes all express the MGP (134). This protein plays a role in preserving the vascular wall's structural and functional integrity (135). It prevents arterial calcifications by binding calcium crystals and blocking bone morphogenetic protein-2 (BMP-2), a pro-mineralizing factor (136). Additionally, MGP preserves the extracellular matrix's composition and inhibits the osteoblastic development of vascular smooth muscle cells (134).

Changes in vitamin K2 metabolism are linked to quantitative changes in the constitute of the gut microbiota, such as in small intestinal bacterial overgrowth (SIBO). Specifically, regardless of daily vitamin K2 intake, individuals with SIBO had considerably greater serum levels of dephosphorylated-uncarboxylated matrix Gla-protein, the inactive form of MGP, than controls. Higher levels of inactive MGP in patients were associated with an increase in arterial stiffness as determined by pulse-wave velocity, which was one of the early indicators of vascular dysfunction (137). One of the most reliable indicators of poor vitamin K2 status is generally thought to be elevated levels of the inactive form of MGP (138). Furthermore, it has been demonstrated that increases in MGP inactive form are linked to early vascular disease symptoms as well as cardiovascular morbidity and mortality (139–143).

## 3 New strategies in CVD prevention and treatment

Researchers have focused on gut microbiota and associated metabolites to prevent and treat CVD. Therefore, as a new regulator of CVD, the gut microbiota has emerged as a possible therapeutic target.

### 3.1 Diet

The composition and function of the gut microbiota can be altered by diet in several ways (144). These changes take time to manifest, and they have more significant impact if they are sustained over time (145). Short-term dietary changes can rapidly and temporarily change the diversity of human microbiota. Long-term dietary patterns influence the development of each person's stable microbiota profile (145, 146). The idea that a regulated diet may result in positive changes in the composition of the gut microbiota in the prevention of CVD is supported by an observational study by Wang et al. (147).

High-fiber diets inhibit the growth of known opportunistic pathogens and promote the growth of helpful commensal bacteria (148, 149). A high-fiber diet has been shown to reduce blood pressure, lessen heart hypertrophy and fibrosis, and boost the microbiota that produces acetate (150). According to Xiao et al., dietary interventions using whole grains and foods from traditional Chinese medicine can enhance intestine beneficial bacteria like *Bifidobacterium* and decrease *Enterobacteriaceae* harmful bacteria (151). Furthermore, a diet rich in fiber can boost the microbiota that produces acetic acid, which reduces blood pressure (150). The quantity of microbiomes that are members of the Bacteroidetes phylum with genera *Prevotella* and *Bacteroides* decreased in CAD patients, and it has been shown that those who eat a high-fiber diet create SCFAs (152). More SCFAs and phosphatidylcholine are produced when the gut bacterial community is modulated by a diet high in plant products, enabling the growth of species that can ferment fibers. A high-fat diet, on the other hand, causes adverse alterations in the fecal metabolomic composition, systemic inflammation, and gut flora (153). Fecal metabolomic patterns are a reflection of changes in the gut bacterial ecology. In fact, a high-fat meal causes circulating pro-inflammatory factors (such as plasminogen activator inhibitor-1, IL-1, and TNF-α mRNA) to rise while SCAs decrease and arachidonic acid and LPS production increases (154). Another study demonstrated the anti-inflammatory effects of a Mediterranean diet, showing a negative relationship between the production of SCFAs and the expression of inflammatory cytokines, including VEGF, MCP-1, IFN-γ-induced protein 10 (IP-10), IL-17, and IL-12. Additionally, *Enterorhabdus*, *Lachnoclostridium*, and *Parabacteroides* were more prevalent in the Mediterranean diet (155). Mediterranean diets high in fruits and vegetables decreased the incidence of heart failure by 70% in randomized controlled studies (156). Lower TMAO levels in both males and females were also associated with consuming a Mediterranean diet (157). Additionally, 200 g vs. 500 g of unprocessed lean red meat per week differed between two diet groups in a 5-week randomized experiment. The TMAO levels were lower in the group that consumed 200 g of red meat per week than in the group that consumed 500 g (158). When Organ et al. performed transverse aortic constriction surgery on C57BL6/J mice to cause HF, they discovered that the animals receiving TMAO or choline-supplemented diets showed worse HF symptoms and biomarkers than the mice receiving a control diet (159). According to another study, a lower ratio of *Firmicutes*, including *Bacteroidetes* and *Streptococcus*, and a higher ratio of *Catenibacterium*, *Bifidobacterium*, and fecal SCFAs were linked to a closer adherence to the Mediterranean dietary pattern and increased consumption of plant-based nutrients, such as vegetable proteins and polysaccharides (160). In contrast to a diet heavy in saturated fat, which can raise LDL cholesterol, a westernized diet high in unsaturated fat can boost Bacteroidetes and decrease *Firmicutes* and *Bilophila wadsworthia* (sulfite-reducing microorganisms) (161).

Sodium chloride, or NaCl, is another name for salt, an essential component of human nutrition. The words “salt” and “sodium” are commonly used in ways that imply they have the same meaning (162). A daily consumption of less than 5 g of salt is advised by the World Health Organization's (WHO) Healthy Diet Fact Sheet (163). Several recent studies have examined the effects of a high salt intake on gut microbiology and disease (164), including inflammatory bowel disease (162) and cardiovascular disease (165), given the well-established relationship between diet and gut microbiology and its implications for the development of disease. Recent research has shown that consuming too much salt, especially sodium, might raise blood pressure by altering the gut flora (164, 166). One example of this is the advice to limit salt consumption because some people and model organisms are sensitive to a diet high in salt (38, 167). A high-salt diet mechanistically increases intestinal permeability and pro-inflammatory cytokines in human and animal research, promoting local and systemic tissue inflammation (164, 168, 169). Certain bacteria, such as *Bacteroides fragilis*, may be responsible for some of the effects of dietary salt. They activate the mineralocorticoid receptor and raise blood pressure through intermediate metabolic actions (170). Ferguson JF and colleagues found that a high-salt diet decreased the number of lactate-producing bacteria, such as those belonging to the Bacilli class, the *Lactobacillales* order, the Leuconostocaceae family, and the Leuconostoc genus, in both people and mice. Usually, these bacteria prevent hypertension and T-cell activation by salt (164, 171). According to a different study by Wilck et al., eating a lot of salt impacts the gut microbiota, mainly by decreasing Lactobacillus murinus and raising Th17 cells and blood pressure. L. murinus supplementation reduced hypertension and inhibited Th17 activation (164).

These studies demonstrate how a diet high in salt can negatively impact gut microbiota, which can then affect gut health and contribute to the development and progression of cardiovascular disease. Given the intricate relationships between food, gut microbiota, and the metabolites that follow, diet is a significant risk factor for CVD and cardiovascular health. To maintain physical health, patients with CVD must optimise their food composition and make suitable dietary adjustments.

### 3.2 Probiotics and prebiotics

A growing body of research is investigating how probiotics can lower cardiovascular risk, particularly in light of recent findings linking the gut microbiota to the etiology of CVD. According to the Greek definition, probiotics are “live microorganisms which, when administered in adequate amounts, confer health benefits on the host” (Pro: promotion, Biotic: life) (172). *Lactobacillus*, *Bifidobacterium*, *Lactococcus*, *Streptococcus*, and *Enterococcus* are common probiotics (173). Across many cultures, people eat a wide variety of fermented foods that contain probiotic strains, including yoghurt, kefir, sauerkraut, tempeh, and kimchi (174). Probiotics may work in a variety of ways, including adjusting pH, producing antibiotic compounds, and competing with pathogens (175). Prebiotics are defined as “non-digestible food components allowing the specificity of microbial changes in the intestinal tract, thereby exhibiting beneficial effects on host's health,” as was initially set there by Gibson and Roberfroid in 1995 and then revised in 2004 (176, 177). The International Scientific Association for Probiotics and Prebiotics has since maintained the most widely recognized definition of this term as a substrate that host bacteria preferentially use to provide health benefits (178). Studies conducted both *in vitro* and *in vivo* have shown that oligosaccharides, particularly fructans [fructooligosaccharides (FOS) and inulin] and galactans [galactooligosaccharides (GOS)], are the most well-known prebiotics (179). The majority of prebiotics are carbohydrates found in foods such as cereals, fruits, and vegetables (173). Probiotics and prebiotics' ability to alter gut microbiota has emerged as a new avenue for CVD prevention and treatment. Numerous studies have demonstrated the positive effects of prebiotics and probiotics on lipid regulation, which has indirect advantages for CVD (180–183). Furthermore, there is strong evidence that functional foods that contain prebiotics and probiotics may help prevent several cardio-metabolic conditions, such as type I diabetes, obesity, and hypertension (184–186). Another explanation for the protective benefits of probiotic and prebiotic treatments on CVD is host immune system regulation. Changes in epithelial cells, dendritic cells, effector lymphocytes, natural killer T cells, T regulatory cells, and B cells are among the immunological processes that probiotics and prebiotics support (187).

Some studies examined the positive benefits of inulin or inulin-containing prebiotics on a range of CVDs in both human and animal models, including coronary artery disease, chronic renal disease, atherosclerosis, hypercholesterolemia, and CHD or diabetes linked to CHD (188–192). Furthermore, it was discovered that the probiotics *Lactobacillus fermentum* and *Bifidobacterium* breve can lower blood pressure by preventing endothelial dysfunction and re-establishing the balance of the gut microbiota (193). In patients with CKD, the treatment of *Lactobacillus sp.* was linked to a considerable decrease in small intestinal toxins such as dimethylamine and nitrosodimethylamine, and in patients with carotid atherosclerosis, it was related to alterations in colon levels of certain SCFAs (194, 195). A 6-week daily supplementation with *Lactobacillus plantarum 299v* (Lp299v) has a positive effect on CVD by causing changes in circulating metabolites originating from the gut microbiome, according to a pilot trial including 21 men with stable coronary artery disease. Lp299v supplementation can decrease systemic inflammation and enhance endothelium-dependent brachial artery vasodilation by increasing nitric oxide bioavailability (196). Additionally, Lam et al. discovered that *Lactobacillus plantarum* could decrease the size of myocardial infarctions and enhance ventricular function (197). One gut microbiota component that has positive impacts on the pathophysiology of cardiovascular disease and arterial hypertension is *Akkermansia muciniphila* (198). Patients with heart failure can benefit from *Saccharomyces boulardii*'s ability to lower serum creatinine and inflammatory marker levels (199). Although probiotics and prebiotics can be added to the diet of patients with CVD based on their circumstances to improve gut dysbiosis and regulate the gut microbiota, there are still unanswered questions regarding the precise immunological and physiological effects they may have on health and illness, necessitating more research.

### 3.3 Fecal microbiota transplantation

Transplanting a fecal solution from a healthy donor into the recipient's digestive tract is known as fecal microbiota transplantation (FMT), and it has been used to treat ulcerative colitis and *Clostridium difficile* infections (200, 201). Stools from healthy donors or recipients (self-FMT) are collected as part of this procedure before being administered to patients with illnesses or associated dysbioses. These complicated investigations about CVD endpoints have not yet been thoroughly examined. Although the clinical usefulness of this strategy for CVD is debatable, FMT has been shown to have a therapeutic effect against small intestine permeability and insulin resistance (202–204). The use of FMT via fecal enema as an adjuvant in treating pseudomembranous colitis was initially reported by Eisman et al. (205). FMT is much more effective than standard treatment procedures, including vancomycin antibiotics, and it has been instrumental in treating recurring *Clostridium difficile* infections (206, 207). Additionally, FMT is promising as a treatment for additional conditions linked to the microbiota, such as Crohn's disease, ulcerative colitis, obesity, type 2 diabetes, and cardiovascular disease.

FMT has also recently been investigated as a potential treatment for cardiometabolic diseases (208, 209). Fecal donation improved myocarditis in animal models (210). To maintain a balanced gut flora, it is thought to support beneficial microorganisms, compete with pathogens, and restore a healthy gut microbiota. Restoring the gut's health could aid in treating CVD because dysbiosis and gut microbiome are linked to the disease. In fact, in experimental mouse models of autoimmune myocarditis, FMT was shown to alleviate myocarditis (210). Increasing the Firmicutes/Bacteroidetes ratio and decreasing inflammatory infiltration are two proposed ways. Through the anti-inflammatory mechanism, the study validates the impact of gut flora on CVD and the significant function of Bacteroidetes in microbiota composition (211). More preclinical research expands our understanding of FMT and its potential by transferring the feces of healthy people and hypertension patients into germ-free mice. As expected, mice that received patient microbiomes had more tremendous blood pressure than mice that received healthy microbiomes (212). Furthermore, the impact of FMT on atherosclerosis was also investigated (213).

In a randomized, double-blind, controlled study with 20 patients with metabolic syndrome, it was discovered that vegetarians' post-single transplant fecal flora might alter the intestinal flora composition of specific individuals but not the vasculitis-related measures (214). Recently, Fan et al. reported their clinical trial design of FMT effects on primary hypertension patients. The expected outcomes include microbiota profile, blood pressure, blood glucose and lipids, ankle-branchial index, and the causes of events. This investigation may be among the earliest clinical trials to examine the effects of FMT on hypertension, offering crucial data for additional research. Patients with hypertension were found to benefit from a modified form of FMT known as the “washed microbiota transplantation effect.” (215).

However, due to the hazards involved, such as the potential for endotoxins or infectious organisms to spread and produce new gastrointestinal issues, the use of FMT is currently restricted (216, 217).

Without preclinical trials of FMT on CVD, including CAD, PAD, and heart failure, clinical trials may not have sufficient data and support to be carried out. Therefore, research on the FMT approach to CVD therapy and prevention is still needed.

### 3.4 Small-molecule antimicrobial enzyme therapeutics

As discussed earlier, gut microbiota produces a metabolite called TMA through the breakdown of dietary phosphatidylcholine/choline, L-carnitine, or betaine in consumed red meat or animal flesh (41, 42, 63, 218). Certain choline TMA lyase inhibitors can help lower plasma TMAO levels and improve atherosclerosis and thrombosis. It has been demonstrated that the natural substance 3,3-dimethyl-1-butanol (DMB), which is present in some red wines, balsamic vinegar, cold-pressed extra virgin olive oils, and grape seed oils, lowers plasma TMAO in mice given a chow diet supplemented with carnitine or choline (218). Furthermore, DMB reduces the *in vivo* rate of thrombus formation and platelet reactivity, which a choline diet improves (219).

By creating a small-molecule tool medication to limit microbial choline TMA lyase activity, this technique recently demonstrated proof of concept (218). Although there is currently little information available, new choline TMA-lyase inhibitors have recently been created, such as iodomethylcholine (IMC) and fluoromethylcholine (FMC) (220). Without prolonging the bleeding period, the IMC and FMC molecules can lower the host's TMA and TMAO levels, which will limit platelet aggregation and the *in vivo* rate of thrombus formation (219). FMO3, another regulator in the production pathway for TMAO, quickly transforms TMA into TMAO. Berberine, 3, 3C-diindolylmethane (DIM), and indole-3-carbinol are examples of phytochemicals that have demonstrated promise in lowering the generation of TMAO and suppressing FMO3 activity (221, 222). Research on the antisense oligonucleotide-based regulation of FMO3 in animal models revealed a decrease in TMAO serum levels concurrent with a reduction in diet-enhanced atherosclerosis (223). Thus, the possibility of developing microbial enzyme inhibitors to treat people's cardiometabolic abnormalities is quite exciting.

## 4 Conclusion

Despite mounting evidence linking gut microbiota to cardiovascular diseases (CVDs), significant information gaps still exist. The absence of clear evidence connecting particular changes in the microbiome to cardiovascular disease is one of the main drawbacks of current research. Since the majority of research uses observational and correlational data, it is challenging to determine whether gut dysbiosis is a direct cause of CVD development or merely an effect of the illness state. These linkages may become clearer in the future with the use of advanced microbiome sequencing, metabolomics, and causal inference techniques like Mendelian randomization.

Inter-individual variation in gut microbiota composition, which is impacted by environmental exposures, genetics, nutrition, and drugs, is a further major challenge. The development of standardized microbiome-based treatments for CVD is complicated by this diversity. Our review highlights the potential for patient-specific microbial profile-based specific gut microbiome therapies, an area that needs more investigation. Furthermore, although a number of metabolites originating from the gut (such as bile acids, SCFAs, and TMAO) have been linked to cardiovascular function, the exact molecular mechanisms behind the gut-heart axis are still not fully known. Finding new microbial metabolites and understanding how they specifically affect inflammation, atherosclerosis, and endothelial function may lead to the discovery of new treatment targets. Finally, there is still difficulty in implementing microbiome-targeted treatments in clinical settings. Although fecal microbiota transplantation (FMT), probiotics, prebiotics, and microbial enzyme inhibitors have demonstrated promise, their long-term safety and effectiveness in preventing and treating CVD are yet unknown. This review addresses new approaches that may help close this gap, such as targeted enzyme inhibitors and precision medicine based on the microbiome. Appropriate treatment approaches that target the gut microbiota promise improved CVD management and prevention in the future.
